# Early hemoglobin trajectories and short-term mortality in ICU patients with traumatic brain injury: development in MIMIC-IV and external assessment in eICU-CRD

**DOI:** 10.3389/fneur.2026.1967355

**Published:** 2026-10-06

**Authors:** Saijie Li, Yanyu Han, Feiyu Ding, Zhichao Qiu, Xuezi Ma, Hanyu Huang, Bin Zhang, Handong Wang

**Affiliations:** 1 Department of Neurosurgery, Nanjing BenQ Medical Center, The Affiliated BenQ Hospital of Nanjing Medical University, Nanjing, China; 2 Nanjing Medical University, Nanjing, China; 3 Department of Infectious Diseases, Affiliated Hospital of Nantong University, Medical School of Nantong University, Nantong, China

**Keywords:** eICU-CRD, external assessment, hemoglobin, latent class linear mixed model, longitudinal trajectory, MIMIC-IV, traumatic brain injury

## Abstract

**Background:**

Hemoglobin (Hb) changes dynamically after traumatic brain injury (TBI), but the prognostic relevance of distinct longitudinal patterns and their added value beyond early Hb remain uncertain. We characterized early Hb trajectories in ICU patients with TBI and examined associations with mortality after a 120-h landmark.

**Methods:**

MIMIC-IV was used for model development and multicenter eICU-CRD for external assessment. Daily median Hb values across five 24-h windows were modeled using a latent class linear mixed model. The three-class MIMIC-IV model was frozen before transport to eICU. Cox models evaluated mortality from 120 h through ICU day 28, with sensitivity analyses addressing selection, landmark attrition, simpler Hb summaries, classification uncertainty, PRBC exposure, missingness, and nonproportional hazards.

**Results:**

In MIMIC-IV, 2,466 patients entered trajectory analysis and 2,409 entered post-landmark analysis. Three trajectories were identified: relatively high and stable, rapidly declining, and persistently low Hb. In fully adjusted models, rapidly declining (HR 1.74, 95% CI 1.18–2.57) and persistently low Hb (HR 1.57, 95% CI 1.19–2.08) were associated with higher mortality. In eICU, 2,097 patients were classified and 1,557 entered outcome analysis; persistently low Hb remained associated with mortality (HR 1.58, 95% CI 1.03–2.43), whereas the rapidly declining association was less precise (HR 1.24, 95% CI 0.80–1.92). Adding trajectory class to the MIMIC-IV clinical model increased the C-index from 0.786 to 0.792. After adjustment for first-day Hb, the rapidly declining trajectory remained associated with mortality (HR 1.77, 95% CI 1.20–2.62), whereas the persistently low association attenuated (HR 1.27, 95% CI 0.83–1.93). Independent three-class refitting in eICU did not recover the same latent-class structure (ARI 0.169).

**Conclusion:**

Early Hb trajectories after TBI showed distinct prognostic associations. Persistently low Hb had the most consistent transported association but was attenuated after first-day Hb adjustment; rapid decline retained an association in MIMIC-IV but had limited external support. Incremental discrimination was modest. These patterns should be interpreted as treatment-embedded clinical courses rather than fixed biological phenotypes or evidence for trajectory-specific transfusion thresholds.

## Introduction

Traumatic brain injury remains an important cause of death and long-term neurological disability worldwide, and its clinical heterogeneity extends far beyond simple classification as mild, moderate, or severe (1, 2). In critically ill patients, a major goal of management is to prevent secondary brain injury, with maintenance of cerebral perfusion and oxygen delivery being particularly important (3). Hemoglobin is a major determinant of arterial oxygen content, but it is not a static marker. Anemia commonly develops early after moderate-to-severe TBI and may be related to extracranial injury, surgery, and volume resuscitation (4). Multicenter data also show that Hb levels and transfusion practices change substantially during the first week after injury (5).

Associations between Hb and adverse outcomes after TBI have been reported in several studies, although the findings have not been entirely consistent. Sekhon et al. found that lower 7-day mean Hb was associated with higher in-hospital mortality in patients with severe TBI (6). Studies using brain tissue oxygen monitoring have also suggested that anemia may increase the risk of cerebral tissue hypoxia (7). A CENTER-TBI analysis showed that lower Hb during the first week was associated with unfavorable 6-month neurological outcomes and mortality (5).

However, Hb levels change dynamically during hospitalization after TBI, and the same initial Hb value may precede very different subsequent courses. At the same time, a latent trajectory should not be assumed to add prognostic information simply because it summarizes repeated measurements. Whether trajectory membership provides information beyond first-day Hb or simpler summaries such as mean, minimum, or slope therefore requires direct evaluation.

Trajectory analysis offers one way to summarize longitudinal heterogeneity without imposing a single average course on all patients. Nevertheless, latent classes are data-driven statistical patterns rather than necessarily discrete biological phenotypes, and their reproducibility can depend on model specification and case mix. In this study, we developed early Hb trajectories during the first 120 h after ICU admission in MIMIC-IV, transported the frozen model to eICU-CRD for external assessment, and examined associations with subsequent short-term mortality. We also compared trajectory classification with simpler Hb representations and evaluated selection, classification uncertainty, treatment during trajectory formation, missingness, and proportional-hazards assumptions.

## Materials and methods

### Study design and data sources

This was a retrospective cohort study using two critical care databases. MIMIC-IV version 3.1 was used as the model development database and contains de-identified hospital and ICU electronic health records from Beth Israel Deaconess Medical Center (8). The eICU Collaborative Research Database version 2.0 served as the multicenter external assessment database and contains de-identified ICU data from multiple hospitals in the United States (9). Both databases were accessed through PhysioNet. The original database studies received appropriate ethical approval and waivers of informed consent, and the present study was conducted in accordance with the corresponding data-use agreements.

Data were extracted using PostgreSQL and processed and analyzed in R version 4.5.3. Latent class linear mixed models were fitted using lcmm version 2.2.2. The model structure and number of classes selected in MIMIC-IV were fixed before analysis of eICU, and outcomes in eICU were not used to reselect the development model. This prespecified transport strategy follows general principles of external assessment, in which the model specification is retained when performance is examined in a new dataset (10). We therefore treated eICU as an assessment of transportability and prognostic association rather than validation of an individual-level prediction model. The distinction between development and external evaluation is also consistent with broader transparent-reporting principles (11), while acknowledging that the present study is not a conventional prediction-model study.

### Study population

Only the first eligible ICU stay for each patient was included. The inclusion criteria were: (1) age ≥18 years; (2) an ICU admission; (3) a clearly documented diagnosis of acute traumatic brain injury (TBI) during the hospitalization; (4) ascertainable vital status; and (5) sufficient early Hb measurements after ICU admission for trajectory modeling. Glasgow Coma Scale (GCS) score was not used as an inclusion threshold to avoid excluding patients with mild-to-moderate TBI who nevertheless required intensive care.

In MIMIC-IV, acute TBI was primarily identified using ICD diagnosis codes, including ICD-9 codes 850–854, ICD-10 codes S06*, and selected ICD-9 skull-fracture subcodes 800–804 whose diagnosis descriptions explicitly contained intracranial injury, concussion, cerebral laceration or contusion, or traumatic intracranial hemorrhage. Sequelae and subsequent-encounter codes were excluded. Because diagnostic information in eICU-CRD is structured differently from MIMIC-IV, adult ICU patients with TBI were identified using codes and diagnostic terms from diagnosis, admissionDx, and related admission diagnosis fields, applying conceptually consistent disease-identification rules across databases.

Exclusion criteria were: (1) isolated skull fracture; (2) unspecified head injury; (3) remote injury or TBI sequelae; (4) post-concussion syndrome; (5) nontraumatic intracranial hemorrhage, stroke, or intracranial tumor; and (6) insufficient Hb measurements during 0–120 h after ICU admission for early trajectory modeling. For patients with multiple ICU admissions, only the first eligible ICU stay was retained to reduce bias from repeated inclusion.

### Hb measurements and trajectory modeling

All available Hb measurements during 0–120 h after ICU admission were collected and divided into five consecutive 24-h windows: 0–24 h, 24–48 h, 48–72 h, 72–96 h, and 96–120 h. All Hb values were converted to g/L. When multiple measurements were available within the same window, the median value was used as the representative value. Trajectory analysis required Hb measurements in at least three of the five windows. Unobserved windows were treated as missing without separate imputation, and the latent class linear mixed model used maximum likelihood estimation to incorporate all available repeated measurements from unbalanced longitudinal data.

Latent class linear mixed models were fitted using the hlme function in the R package lcmm (12). A one-class model was first fitted to obtain stable initial values, after which linear and quadratic time functions with two to five classes were compared. Because only five prespecified time points were available, higher-order polynomial functions were not considered. Multiclass models were fitted using repeated searches from multiple initial values and were required to converge normally with identifiable parameters.

The final model was selected by jointly considering log-likelihood, Akaike information criterion (AIC), Bayesian information criterion (BIC), normalized entropy, average posterior probability of assignment (APPA), odds of correct classification (OCC), minimum class proportion, convergence, and trajectory shape (13–15). APPA ≥0.70 and OCC > 5 were treated as supportive indicators rather than sole selection criteria. To avoid clinically sparse solutions, models producing a class <5% were deprioritized. Thus, although lower BIC values were observed for some higher-class solutions, the three-class quadratic model was retained as the most balanced solution across statistical fit, classification adequacy, and class size.

Internal stability was evaluated using 100 patient-level bootstrap resamples. The adjusted Rand index (ARI) and patient-level class concordance after refitting were recorded, including their distributions rather than only central estimates. Patients in the development cohort were assigned to the class with the highest posterior probability, while the full posterior-probability vector was retained for classification-uncertainty analyses.

For the primary external assessment, the frozen three-class quadratic MIMIC-IV model was directly applied to Hb data in the same five time windows in eICU, and patients were assigned according to model-estimated posterior class probabilities. The transported class definitions were not altered according to eICU outcomes. As a complementary analysis, a three-class quadratic model was independently refitted in the eICU trajectory cohort. Because latent-class labels are arbitrary, the local refit was interpreted using its five-window predicted Hb profiles, and patient-level partition similarity with the transported classification was quantified using the label-invariant ARI. We did not treat numerical label agreement alone as evidence that the same clinical trajectory structure had been recovered.

### Outcome definition and start of follow-up

Trajectory exposure was constructed using Hb information from 0 to 120 h after ICU admission. ICU hour 120 was used as a landmark so that trajectory formation preceded outcome observation. The primary survival risk set therefore included only patients who were alive and remained observable with positive follow-up time at 120 h. Death before 120 h, live discharge before 120 h, and any other reason for lack of post-landmark observation were tabulated separately. Consequently, post-landmark hazard ratios apply to patients who survived and remained observable at the 120-h landmark rather than to all ICU patients with TBI.

The primary MIMIC-IV outcome was all-cause mortality from 120 h after ICU admission through ICU day 28, with patients who remained alive administratively censored at day 28. Because eICU lacks reliable post-discharge mortality information, the external outcome was in-hospital mortality during the same time interval; patients discharged alive were censored at hospital discharge and those remaining hospitalized beyond day 28 were administratively censored at day 28. Because the outcome scope differed between databases, no pooled cross-cohort HR was calculated. An additional endpoint-aligned analysis was performed in MIMIC-IV using the same in-hospital death definition and discharge-censoring rule as in eICU.

### Covariate definitions and confounding control

Covariates were prespecified on the basis of their relevance to TBI prognosis, clinical interpretability, temporal ordering, and availability in the databases. They were not selected using univariable *p* values or stepwise regression. The fully adjusted MIMIC-IV model included age, sex, early GCS, non-neurologic SOFA score, mechanical ventilation within 0–24 h, vasoactive drug use within 0–24 h, neurosurgical or neurocritical procedures, and Charlson Comorbidity Index (CCI), thereby covering demographic characteristics, severity of neurologic injury, non-neurologic organ dysfunction, early life support, and comorbidity burden.

The fully adjusted eICU model was limited to variables that could be harmonized with reasonable reliability across databases: age, sex, GCS, mechanical ventilation within 0–24 h, and vasopressor use within 0–24 h. Median Hb during 0–24 h was part of the trajectory exposure and was not included in the primary model. Variables with substantial missingness, including BMI and lactate, were used descriptively and were not included in the primary adjusted models. Early packed red blood cell (PRBC) transfusion occurred during the trajectory-construction window and could both respond to preceding Hb changes and modify subsequent Hb measurements. It was therefore regarded as part of the evolving treatment-embedded clinical course rather than a conventional baseline confounder.

### Statistical analysis

Continuous variables are presented as medians with interquartile ranges (IQRs), and categorical variables as *n* (%). Survival probabilities across trajectory groups were described using Kaplan–Meier curves and compared overall using the log-rank test.

Three Cox proportional hazards models were fitted in MIMIC-IV: model 1 was unadjusted, model 2 adjusted for age and sex, and model 3 additionally adjusted for the prespecified clinical covariates. Corresponding models were fitted in eICU. Because patients were clustered within hospitals, robust standard errors clustered by hospital identifier were used for eICU model 3. Results are reported as hazard ratios (HRs), 95% confidence intervals (CIs), and two-sided *p* values, with the relatively high and stable Hb trajectory as the reference group.

Primary Cox models were fitted using complete cases, and the actual sample size and number of events were reported. The proportional hazards (PH) assumption was assessed using Schoenfeld residuals and cox.zph for the overall model and the trajectory term. In eICU, covariates showing nonproportionality were handled with stratified baseline hazards. For MIMIC-IV, an additional PH-corrected model stratified on binary covariates showing nonproportionality and allowed time-varying effects for continuous covariates when indicated, while retaining a time-constant trajectory effect because the trajectory term itself did not violate the PH assumption.

Additional analyses were performed to address the main sources of uncertainty. First, source-eligible patients who did and did not enter trajectory analysis were compared with respect to early clinical features, ICU length of stay, Hb measurement frequency, and observed-window coverage. Stabilized inverse-probability-of-selection weights were estimated from early covariates and used in weighted Cox sensitivity analyses; P1/P99 winsorization was examined to assess sensitivity to extreme weights. Second, reasons for exclusion before the 120-h landmark were separated and characteristics of landmark-included and landmark-excluded patients were compared.

To examine whether trajectory classification added information beyond simpler Hb representations, we compared a common-sample clinical model with models adding first-day Hb, 0–120-h mean Hb, minimum Hb, patient-specific linear Hb slope, trajectory class, or first-day Hb plus trajectory class. Model fit was summarized using AIC and Harrell’s C-index; changes in C-index relative to the clinical model were evaluated using 200 patient-level bootstrap resamples. Classification uncertainty was examined by repeating model 3 among patients with maximum posterior probability ≥0.70 and ≥0.80 and by 100 pseudo-class draws in which class membership was sampled from each patient’s posterior probabilities.

Treatment-related sensitivity analyses excluded patients with documented PRBC transfusion during 0–120 h and described the timing of first PRBC exposure by trajectory; these analyses were not intended to estimate the causal effect of transfusion. Missingness of primary eICU adjustment variables was quantified, and multiple imputation by chained equations with 20 imputed datasets was used as a sensitivity analysis, with hospital-cluster robust Cox estimates combined using Rubin’s rules. The MIMIC-IV endpoint-aligned analysis, eICU hospital-contribution assessment, and bootstrap stability analysis were retained. All tests were two-sided, with *p* < 0.05 used as a conventional threshold; interpretation emphasized effect estimates and confidence intervals rather than statistical significance alone.

## Results

### Baseline characteristics

The source-eligible cohorts comprised 3,544 MIMIC-IV and 4,380 eICU patients. Of these, 2,466 and 2,097, respectively, had Hb measurements in at least three of five 24-h windows and entered trajectory analysis. Patients entering trajectory analysis had more frequent Hb sampling and longer ICU stays than those excluded for insufficient window coverage. In MIMIC-IV, included versus excluded patients had a median of 5 versus 2 Hb measurements, 5 versus 1 observed windows, and ICU length of stay of 2.9 versus 1.0 days. In eICU, the corresponding values were 5 versus 1 Hb measurements, 4 versus 1 observed windows, and 3.8 versus 1.0 days. Mechanical ventilation and documented PRBC exposure were also more frequent among included patients, consistent with informative measurement and clinical observation (Supplementary Tables S3–S5; Supplementary Figure S1).

Among the 2,466 MIMIC-IV trajectory patients, 57 (2.3%) died before 120 h and 2,409 entered the landmark outcome analysis. Among the 2,097 eICU trajectory patients, 469 (22.4%) were discharged alive before 120 h and 71 (3.4%) died before 120 h, leaving 1,557 patients in the landmark analysis. Thus, the larger pre-landmark attrition in eICU was driven predominantly by early live discharge rather than early mortality (Figure 1; Supplementary Table S12).

**Figure 1:**
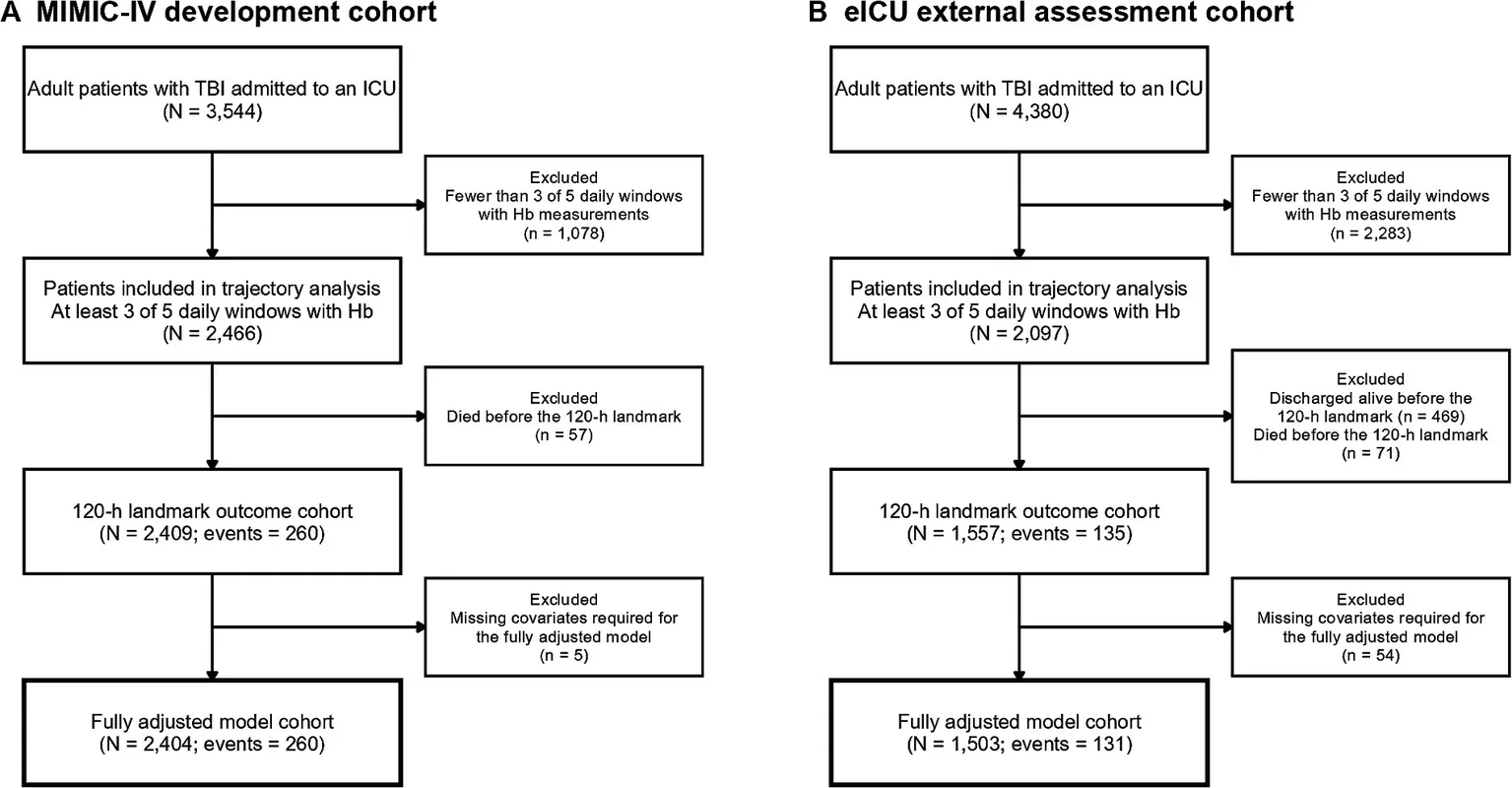
Analytical flow of the MIMIC-IV development cohort and eICU-CRD multicenter external assessment cohort. Panel **(A)** shows cohort construction for the MIMIC-IV development cohort, and panel **(B)** shows cohort construction for the eICU-CRD external assessment cohort. Hb trajectories were constructed from repeated measurements during 0–120 h after ICU admission. The landmark outcome analysis began at 120 h and therefore included only patients who survived and remained observable at that time; reasons for pre-landmark exclusion are reported separately.

In both cohorts, the rapidly declining Hb trajectory was characterized by younger age, a higher proportion of men, and more frequent use of mechanical ventilation, vasopressors, neurosurgical or neurocritical procedures, and PRBC support. The persistently low group was older and had lower early mean arterial pressure and platelet counts; comorbidity burden was higher in MIMIC-IV. Other severity measures, including GCS, differed between databases and were not assumed to show the same between-class pattern (Tables 1, 2). Group *p* values were descriptive only and were not used for covariate selection.

**Table 1:** Baseline characteristics of the MIMIC-IV trajectory cohort according to Hb trajectory class.

Characteristic	Overall	Relatively high and stable Hb	Rapidly declining Hb	Persistently low Hb	*p* value
Demographic characteristics					
Age, years	69.3 [52.7, 82.4]	71.4 [55.6, 82.8]	53.8 [33.2, 69.0]	72.5 [57.7, 84.7]	<0.001
Male sex	1,546 (62.7%)	877 (66.2%)	295 (72.7%)	374 (50.9%)	<0.001
White	1,571 (63.7%)	890 (67.2%)	203 (50.0%)	478 (65.0%)	<0.001
Black	129 (5.2%)	80 (6.0%)	12 (3.0%)	37 (5.0%)	
Asian	62 (2.5%)	38 (2.9%)	7 (1.7%)	17 (2.3%)	
Hispanic	71 (2.9%)	44 (3.3%)	9 (2.2%)	18 (2.4%)	
Other	633 (25.7%)	273 (20.6%)	175 (43.1%)	185 (25.2%)	
Body mass index, kg/m2	26.1 [22.9, 29.7]	25.9 [23.1, 29.7]	26.8 [24.0, 30.9]	24.9 [22.0, 28.9]	<0.001
Clinical severity and support					
Glasgow coma scale score	14.0 [13.0, 15.0]	14.0 [12.0, 15.0]	15.0 [13.0, 15.0]	14.0 [13.0, 15.0]	<0.001
SOFA score	3.0 [2.0, 5.0]	3.0 [2.0, 4.0]	4.0 [2.0, 5.0]	4.0 [2.0, 6.0]	<0.001
Non-neurologic SOFA score	2.0 [1.0, 4.0]	1.0 [1.0, 3.0]	2.0 [1.0, 5.0]	3.0 [1.0, 5.0]	<0.001
Charlson comorbidity index	4.0 [1.0, 6.0]	4.0 [2.0, 5.0]	2.0 [0.0, 4.0]	5.0 [2.0, 7.0]	<0.001
Minimum mean arterial pressure, mmHg	63.0 [56.0, 71.0]	65.0 [58.0, 73.0]	63.0 [56.0, 70.0]	59.0 [52.0, 66.0]	<0.001
Mechanical ventilation within 24 h	1,010 (41.0%)	400 (30.2%)	299 (73.6%)	311 (42.3%)	<0.001
Vasopressor use within 24 h	288 (11.7%)	65 (4.9%)	104 (25.6%)	119 (16.2%)	<0.001
Neurosurgery or neurocritical procedure	510 (20.7%)	247 (18.6%)	122 (30.0%)	141 (19.2%)	<0.001
Admission laboratory values					
Hemoglobin, median within 24 h, g/L	112.0 [98.0, 125.0]	120.5 [111.5, 130.0]	119.0 [110.0, 128.5]	91.0 [83.0, 97.5]	<0.001
Maximum white blood cell count, K/uL	10.8 [7.9, 14.1]	10.2 [7.8, 13.1]	14.2 [11.5, 18.3]	10.0 [7.1, 13.7]	<0.001
Minimum platelet count, K/uL	179.0 [135.0, 228.0]	190.0 [149.0, 238.0]	172.5 [136.5, 227.5]	152.0 [105.2, 207.0]	<0.001
Maximum creatinine, mg/dL	0.9 [0.7, 1.2]	0.9 [0.7, 1.1]	0.9 [0.8, 1.2]	1.0 [0.7, 1.4]	<0.001
Minimum sodium, mmol/L	139.0 [136.0, 141.0]	139.0 [136.0, 141.0]	139.0 [136.0, 141.0]	138.0 [136.0, 141.0]	0.357
Maximum sodium, mmol/L	140.0 [138.0, 143.0]	140.0 [137.0, 142.0]	141.0 [139.0, 144.0]	140.0 [137.0, 143.0]	<0.001
Maximum INR	1.2 [1.1, 1.3]	1.2 [1.1, 1.3]	1.2 [1.1, 1.3]	1.2 [1.1, 1.4]	<0.001
Maximum lactate, mmol/L	2.0 [1.3, 3.3]	1.7 [1.2, 2.4]	2.5 [1.6, 3.7]	2.0 [1.3, 3.5]	<0.001
TBI injury patterns (non-mutually exclusive)					
Skull fracture	627 (25.4%)	293 (22.1%)	173 (42.6%)	161 (21.9%)	<0.001
Epidural hematoma	384 (15.6%)	185 (14.0%)	94 (23.2%)	105 (14.3%)	<0.001
Subdural hematoma	1,577 (63.9%)	883 (66.6%)	241 (59.4%)	453 (61.6%)	0.008
Traumatic subarachnoid hemorrhage	509 (20.6%)	257 (19.4%)	93 (22.9%)	159 (21.6%)	0.227
Traumatic intracerebral/intraparenchymal hemorrhage	15 (0.6%)	7 (0.5%)	3 (0.7%)	5 (0.7%)	0.825
Cerebral contusion/laceration	590 (23.9%)	249 (18.8%)	147 (36.2%)	194 (26.4%)	<0.001
Early blood support					
PRBC transfusion within 120 h	388 (15.7%)	34 (2.6%)	112 (27.6%)	242 (32.9%)	<0.001
Outcome					
Post-landmark all-cause death through ICU Day 28	260 (10.8%)	101 (7.7%)	40 (10.3%)	119 (16.8%)	<0.001

Continuous variables are presented as median [IQR] and categorical variables as *n* (%). Percentages were calculated using the actual nonmissing denominator. Group *p* values were not used for covariate selection.

**Table 2:** Baseline characteristics of the eICU-CRD multicenter external assessment cohort according to the MIMIC-IV-derived Hb trajectory classes.

Characteristic	Overall	Relatively high and stable Hb	Rapidly declining Hb	Persistently low Hb	*p* value
Demographic characteristics					
Age, years	64.0 [46.0, 79.0]	68.0 [51.0, 81.0]	51.0 [31.0, 66.0]	68.0 [50.0, 82.0]	<0.001
Male sex	1,294 (61.7%)	736 (62.2%)	271 (71.9%)	287 (53.4%)	<0.001
Missing/not reported	16 (0.8%)	9 (0.8%)	2 (0.5%)	5 (0.9%)	0.327
African American	157 (7.5%)	85 (7.2%)	28 (7.4%)	44 (8.2%)	
Asian	55 (2.6%)	33 (2.8%)	5 (1.3%)	17 (3.2%)	
Caucasian	1,637 (78.1%)	921 (77.9%)	293 (77.7%)	423 (78.8%)	
Hispanic	92 (4.4%)	57 (4.8%)	15 (4.0%)	20 (3.7%)	
Native American	20 (1.0%)	10 (0.8%)	3 (0.8%)	7 (1.3%)	
Other/Unknown	120 (5.7%)	68 (5.7%)	31 (8.2%)	21 (3.9%)	
Body mass index, kg/m2	25.9 [22.8, 30.0]	25.9 [22.8, 29.9]	26.6 [23.5, 30.9]	25.7 [22.1, 29.5]	0.006
Clinical severity and support					
Glasgow coma scale score	13.0 [7.0, 15.0]	14.0 [10.0, 15.0]	8.0 [4.0, 14.0]	12.0 [7.0, 15.0]	<0.001
APACHE score	53.0 [39.8, 71.0]	47.0 [36.0, 60.0]	64.0 [44.0, 79.0]	60.0 [48.0, 81.0]	<0.001
Acute physiology score	38.0 [27.0, 58.0]	33.0 [25.0, 47.0]	55.0 [35.0, 74.0]	47.0 [34.0, 68.0]	<0.001
Minimum mean arterial pressure, mmHg	63.0 [54.0, 72.0]	66.0 [57.0, 74.0]	62.0 [52.8, 70.0]	59.0 [50.0, 67.8]	<0.001
Mechanical ventilation within 24 h	959 (45.7%)	395 (33.4%)	283 (75.1%)	281 (52.3%)	<0.001
Vasopressor use within 24 h	175 (8.3%)	45 (3.8%)	65 (17.2%)	65 (12.1%)	<0.001
Neurosurgery or neurocritical procedure	282 (13.4%)	120 (10.1%)	81 (21.5%)	81 (15.1%)	<0.001
Admission laboratory values					
Hemoglobin, median within 24 h, g/L	114.0 [100.0, 129.0]	123.0 [112.4, 134.0]	119.0 [109.0, 132.0]	91.0 [83.0, 97.0]	<0.001
Maximum white blood cell count, K/uL	11.1 [8.2, 14.7]	10.3 [7.8, 13.4]	14.0 [10.7, 17.8]	10.8 [7.7, 14.6]	<0.001
Minimum platelet count, K/uL	183.0 [135.0, 228.0]	193.0 [154.0, 233.0]	173.0 [136.0, 223.0]	152.0 [105.0, 215.0]	<0.001
Maximum creatinine, mg/dL	0.9 [0.7, 1.1]	0.8 [0.7, 1.0]	0.9 [0.8, 1.2]	0.9 [0.7, 1.4]	<0.001
Minimum sodium, mmol/L	139.0 [136.0, 141.0]	138.0 [136.0, 141.0]	139.0 [137.0, 142.0]	139.0 [136.0, 142.0]	<0.001
Maximum sodium, mmol/L	140.0 [137.0, 143.0]	140.0 [137.0, 142.0]	141.0 [139.0, 144.0]	140.5 [137.0, 143.0]	<0.001
Maximum INR	1.2 [1.1, 1.3]	1.1 [1.0, 1.3]	1.2 [1.1, 1.3]	1.3 [1.1, 1.5]	<0.001
Maximum lactate, mmol/L	2.2 [1.3, 3.6]	1.8 [1.1, 2.8]	2.7 [1.8, 3.8]	2.5 [1.4, 4.1]	<0.001
TBI injury patterns (non-mutually exclusive)					
Skull fracture	225 (10.7%)	109 (9.2%)	67 (17.8%)	49 (9.1%)	<0.001
Epidural hematoma	89 (4.2%)	40 (3.4%)	16 (4.2%)	33 (6.1%)	0.031
Subdural hematoma	1,020 (48.6%)	571 (48.3%)	169 (44.8%)	280 (52.1%)	0.087
Traumatic subarachnoid hemorrhage	676 (32.2%)	365 (30.9%)	133 (35.3%)	178 (33.1%)	0.242
Traumatic intracerebral/intraparenchymal hemorrhage	360 (17.2%)	214 (18.1%)	60 (15.9%)	86 (16.0%)	0.444
Cerebral contusion/laceration	165 (7.9%)	66 (5.6%)	47 (12.5%)	52 (9.7%)	<0.001
Early blood support					
PRBC treatment documented within 120 h	112 (5.3%)	14 (1.2%)	30 (8.0%)	68 (12.7%)	<0.001
Outcome					
Post-landmark in-hospital death by ICU Day 28	135 (8.7%)	46 (5.7%)	35 (10.8%)	54 (12.5%)	<0.001

eICU blood-product treatment entries are reported descriptively only and were not considered dose-validated transfusion exposures.

### Development and external application of the Hb trajectory model

Quadratic time functions generally provided better fit than linear functions. Although the four- and five-class quadratic models had lower BIC values, their smallest classes represented only 1.6 and 3.1% of patients, respectively. The three-class quadratic solution was therefore retained as a balance of fit, classification adequacy, and class size (Supplementary Table S1). Posterior classification metrics for the development and transported external classifications are provided in Supplementary Table S2. All 100 bootstrap refits completed successfully. The median ARI was 0.86 (IQR 0.79–0.90; range 0.34–0.96), and median class concordance was 0.95 (range 0.74–0.99), indicating generally stable but not invariant classification across resamples (Supplementary Table S6).

In MIMIC-IV, the relatively high and stable, rapidly declining, and persistently low Hb trajectories accounted for 53.7, 16.5, and 29.8% of patients, respectively (Figure 2A). Median Hb during 0–24 h was similar in the first two groups (120.5 vs. 119.0 g/L), but the rapidly declining trajectory fell to 98.0 g/L during 24–48 h and subsequently remained at approximately 89–92 g/L. The persistently low trajectory started at 91.0 g/L and remained at approximately 86–87 g/L over the five-day period.

**Figure 2:**
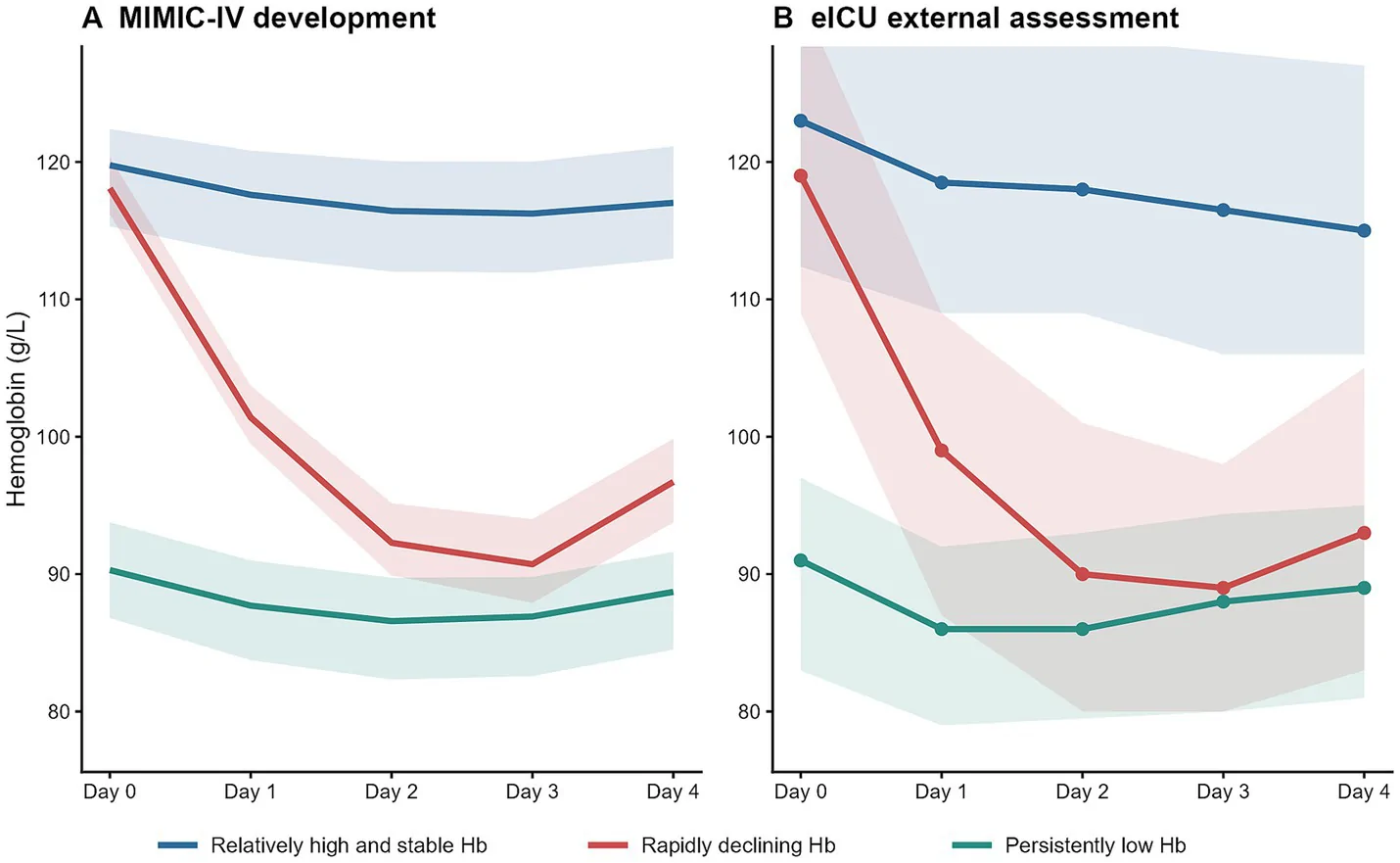
Early five-day Hb trajectories in the development and multicenter external assessment cohorts. Panel **A** shows the predicted trajectories and 95% CIs from the three-class quadratic MIMIC-IV model; panel **B** shows the observed daily median Hb values and IQRs among eICU patients classified by the transported model.

After application of the prespecified MIMIC-IV model to eICU, the three classes accounted for 56.4, 18.0, and 25.6% of patients, respectively, which was close to the distribution in the development cohort (Supplementary Figure S4). The observed daily Hb profiles preserved the same relative ordering and direction of change: the high-stable trajectory declined gradually, the rapidly declining trajectory shifted downward markedly during the first 48 h, and the persistently low trajectory remained low throughout (Figure 2B; Table 2).

### Hb trajectory classes and mortality risk

In MIMIC-IV, survival probabilities from the 120-h landmark differed across the three trajectory groups (log-rank *p* < 0.001). Mortality was 7.7, 10.3, and 16.8% in the relatively high and stable, rapidly declining, and persistently low Hb groups, respectively (Figure 3A).

**Figure 3:**
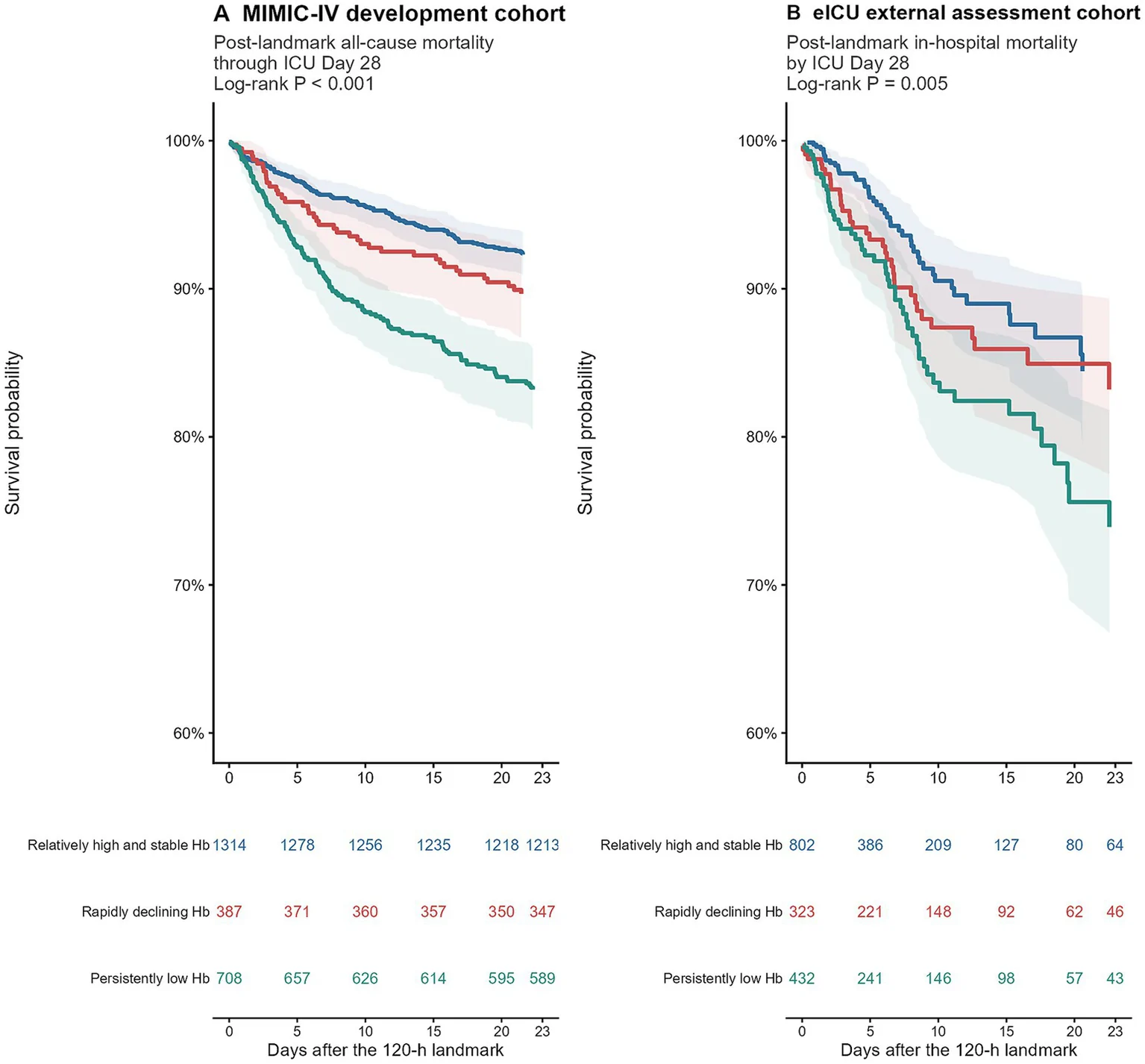
Kaplan–Meier survival curves and numbers at risk from the 120-h landmark after ICU admission. Panel **A** shows all-cause mortality in MIMIC-IV through ICU day 28; panel **B** shows in-hospital mortality over the same time interval in eICU, with patients censored at live hospital discharge. Shaded areas indicate 95% CIs.

Using the relatively high and stable Hb trajectory as the reference, both the rapidly declining Hb trajectory (HR 1.74, 95% CI 1.18–2.57, *p* = 0.005) and the persistently low Hb trajectory (HR 1.57, 95% CI 1.19–2.08, *p* = 0.001) were associated with higher post-120-h mortality risk in fully adjusted model 3. Complete results from the sequentially adjusted models are shown in Table 3 and Figure 4.

**Table 3:** Hb trajectories and mortality risk from the 120-h landmark after ICU admission: Cox regression results.

Cohort	Model	Trajectory	HR	95% CI	*p* value	N/events
MIMIC-IV development	Model 1	Relatively high and stable Hb	1.00	Reference		2,409/260
MIMIC-IV development	Model 1	Rapidly declining Hb	1.37	0.95–1.97	0.095	2,409/260
MIMIC-IV development	Model 1	Persistently low Hb	2.31	1.77–3.01	<0.001	2,409/260
MIMIC-IV development	Model 2	Relatively high and stable Hb	1.00	Reference		2,409/260
MIMIC-IV development	Model 2	Rapidly declining Hb	2.28	1.57–3.32	<0.001	2,409/260
MIMIC-IV development	Model 2	Persistently low Hb	2.27	1.73–2.96	<0.001	2,409/260
MIMIC-IV development	Model 3	Relatively high and stable Hb	1.00	Reference		2,404/260
MIMIC-IV development	Model 3	Rapidly declining Hb	1.74	1.18–2.57	0.005	2,404/260
MIMIC-IV development	Model 3	Persistently low Hb	1.57	1.19–2.08	0.001	2,404/260
eICU external assessment	Model 1	Relatively high and stable Hb	1.00	Reference		1,557/135
eICU external assessment	Model 1	Rapidly declining Hb	1.34	0.86–2.08	0.199	1,557/135
eICU external assessment	Model 1	Persistently low Hb	1.90	1.23–2.94	0.004	1,557/135
eICU external assessment	Model 2	Relatively high and stable Hb	1.00	Reference		1,556/135
eICU external assessment	Model 2	Rapidly declining Hb	1.78	1.15–2.75	0.010	1,556/135
eICU external assessment	Model 2	Persistently low Hb	2.03	1.33–3.12	0.001	1,556/135
eICU external assessment	Model 3	Relatively high and stable Hb	1.00	Reference		1,503/131
eICU external assessment	Model 3	Rapidly declining Hb	1.24	0.80–1.92	0.340	1,503/131
eICU external assessment	Model 3	Persistently low Hb	1.58	1.03–2.43	0.036	1,503/131

Stratified Cox results for eICU are shown in Supplementary Table S7. No pooled cross-cohort HR was calculated.

**Figure 4:**
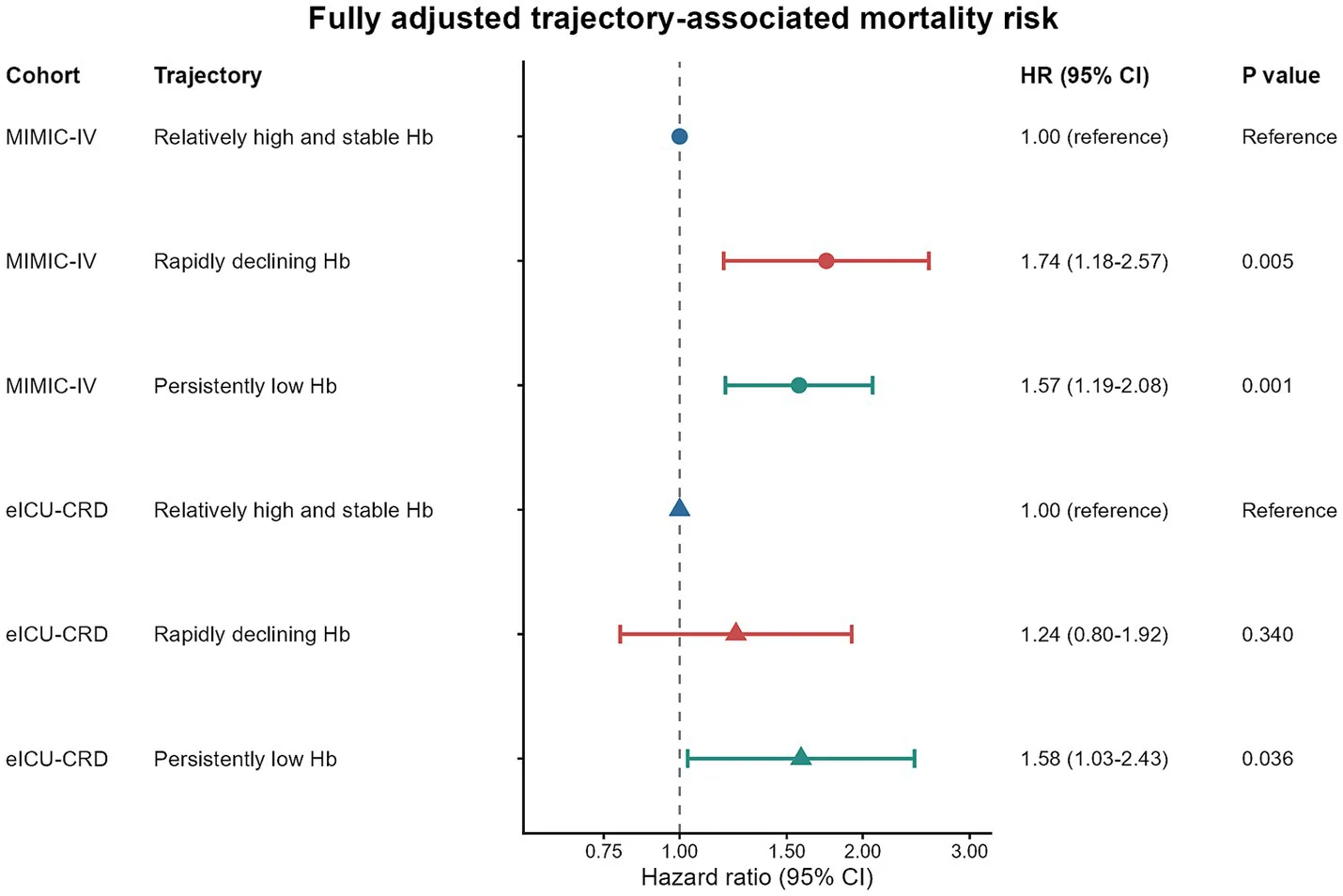
Associations between Hb trajectories and mortality from the 120-h landmark in fully adjusted models across the two cohorts. The relatively high and stable Hb trajectory is the reference. The MIMIC-IV outcome was all-cause mortality through ICU day 28; the eICU outcome was in-hospital mortality over the same interval, with hospital-clustered robust standard errors.

In eICU, in-hospital survival probabilities also differed across the three groups (log-rank *p* = 0.005). In-hospital mortality was 5.7, 10.8, and 12.5% in the relatively high and stable, rapidly declining, and persistently low Hb groups, respectively (Figure 3B).

In fully adjusted model 3 using hospital-clustered robust standard errors, the persistently low Hb trajectory remained associated with higher mortality risk (HR 1.58, 95% CI 1.03–2.43, *p* = 0.036). The point estimate for the rapidly declining Hb trajectory remained greater than 1, but the confidence interval crossed 1 (HR 1.24, 95% CI 0.80–1.92, *p* = 0.340). Thus, the persistently low Hb trajectory received clearer external support, whereas the rapidly declining trajectory showed only directional consistency (Table 3; Figure 4).

### External assessment and sensitivity analyses

In the MIMIC-IV development model, normalized entropy was 0.6517; in the transported eICU classification it was 0.6521, with both values rounding to 0.652 at three decimals. In eICU, APPA ranged from 0.773 to 0.880 and OCC from 5.69 to 22.29. In the transported persistently low group, the median maximum posterior probability was 0.805 and 49.2% of patients had a maximum probability <0.80, indicating meaningful classification uncertainty despite acceptable APPA and OCC values (Table 4).

**Table 4:** Trajectory-model performance and classification quality in the MIMIC-IV development model and eICU external classification.

Cohort	Trajectory	*N*	Percent	APPA	OCC	Median maximum posterior	Maximum posterior <0.70, %	Maximum posterior <0.80, %	Normalized entropy
Panel A: MIMIC-IV frozen model									
MIMIC-IV development	Persistently low Hb	735	29.8	0.785	8.59	0.822	27.5	45.6	0.652
MIMIC-IV development	Relatively high and stable Hb	1,325	53.7	0.887	6.76	0.960	14.1	22.6	0.652
MIMIC-IV development	Rapidly declining Hb	406	16.5	0.812	21.89	0.861	28.6	42.9	0.652
Panel B: eICU transported classification									
eICU external assessment	Rapidly declining Hb	377	18.0	0.830	22.29	0.906	27.6	36.3	0.652
eICU external assessment	Persistently low Hb	537	25.6	0.773	9.88	0.805	32.0	49.2	0.652
eICU external assessment	Relatively high and stable Hb	1,183	56.4	0.880	5.69	0.955	15.5	23.9	0.652

APPA denotes average posterior probability of assignment, and OCC denotes odds of correct classification. Normalized entropy is a model-level measure and was 0.6517 in the MIMIC-IV development model and 0.6521 in the transported eICU classification; both round to 0.652 at three decimal places. Independent eICU refitting is described in Supplementary Table S9 and Supplementary Figure S5.

Independent three-class refitting in the same 2,097 eICU patients did not fully reproduce the MIMIC-IV latent-class structure. One local trajectory closely resembled the rapidly declining pattern, whereas two local trajectories were closer to the relatively high and stable pattern; no distinct local trajectory closely resembled the persistently low Hb pattern. Patient-level partition similarity between the transported and independently refitted classifications was limited (ARI 0.169) (Supplementary Table S9 and Supplementary Figure S5).

In MIMIC-IV model 3, the global PH test was significant (*p* = 0.0029), whereas the trajectory term itself was not (*p* = 0.849). A PH-corrected sensitivity model that stratified on mechanical ventilation and neurosurgical/neurocritical procedures and allowed time-varying effects for age and CCI produced virtually unchanged trajectory estimates: HR 1.74 (95% CI 1.18–2.56) for rapidly declining Hb and HR 1.57 (95% CI 1.19–2.08) for persistently low Hb. In eICU, stratification for covariates with evidence of nonproportionality yielded HRs of 1.32 (95% CI 0.85–2.04) and 1.67 (95% CI 1.09–2.57), respectively (Supplementary Table S7). The corresponding eICU *p* values were 0.218 and 0.018, and the global PH test for the stratified model was *p* = 0.784.

The single largest eICU hospital contributed 5.5% of the trajectory cohort, arguing against domination by a small number of centers. When MIMIC-IV was reanalyzed using the eICU-aligned in-hospital mortality endpoint with live-discharge censoring, the persistently low trajectory remained associated with higher risk (HR 1.57, 95% CI 1.10–2.24), whereas the rapidly declining trajectory was attenuated (HR 1.00, 95% CI 0.61–1.64) (Supplementary Table S8 and Supplementary Figure S7).

Selection-weighted analyses gave estimates close to the primary models. In MIMIC-IV, stabilized inverse-probability-of-selection weighting yielded HRs of 1.75 (95% CI 1.20–2.56) for rapidly declining Hb and 1.57 (95% CI 1.18–2.09) for persistently low Hb. In eICU, the corresponding HRs were 1.26 (95% CI 0.81–1.96) and 1.59 (95% CI 1.04–2.44). Weight distributions were not extreme, and P1/P99 winsorization produced essentially the same estimates (Supplementary Table S11; Supplementary Figure S2).

On a common MIMIC-IV sample of 2,378 patients with 255 events, the clinical model had a C-index of 0.786. Adding trajectory class increased the C-index to 0.792 (bootstrap median ΔC-index 0.0054, 95% interval 0.0005–0.0123), indicating modest incremental discrimination. When first-day Hb and trajectory class were included together, the rapidly declining trajectory remained associated with mortality (HR 1.77, 95% CI 1.20–2.62), whereas the persistently low association was attenuated (HR 1.27, 95% CI 0.83–1.93). In eICU, C-index changes from adding trajectory information were also modest and bootstrap intervals crossed zero (Supplementary Table S13; Supplementary Figure S3).

Results were also broadly consistent when classification uncertainty was considered. Among patients with maximum posterior probability ≥0.80, the MIMIC-IV HRs were 1.76 for rapidly declining Hb and 1.56 for persistently low Hb; the corresponding eICU HRs were 1.21 and 2.22. Across 100 pseudo-class draws, the median HRs were 1.39 and 1.41 in MIMIC-IV and 1.10 and 1.42 in eICU, respectively, again showing less consistent external evidence for the rapidly declining trajectory (Supplementary Table S14).

Among patients with documented PRBC exposure, first transfusions occurred predominantly during the first 24 h, particularly in the rapidly declining and persistently low groups (Supplementary Table S10; Supplementary Figure S6). After excluding patients with PRBC exposure during 0–120 h, the MIMIC-IV HRs were 1.87 (95% CI 1.22–2.88) for rapidly declining Hb and 1.73 (95% CI 1.27–2.35) for persistently low Hb. In eICU patients without documented PRBC treatment, the corresponding HRs were 1.39 (95% CI 0.89–2.17) and 1.50 (95% CI 0.94–2.40). Missingness in the primary eICU model 3 covariates was limited: GCS was missing in 3.4% of patients, sex in 0.06%, and age, mechanical ventilation, and vasopressor use were complete. Finally, multiple imputation of the limited missingness in eICU model 3 covariates yielded HRs of 1.33 (95% CI 0.85–2.07) and 1.63 (95% CI 1.06–2.51), respectively, consistent with the complete-case interpretation (Supplementary Tables S15, S16).

## Discussion

This study yielded four main observations. First, three early Hb patterns were identified in MIMIC-IV and could be assigned in eICU using the frozen development model, but independent refitting in eICU did not recover the same three-class latent structure. Second, persistently low Hb showed the most consistent transported mortality association across the primary MIMIC-IV and eICU analyses, although its association was substantially attenuated after adjustment for first-day Hb. Third, the rapidly declining trajectory began at an Hb level similar to the high-stable group and remained associated with mortality after adjustment for first-day Hb in MIMIC-IV, supporting a dynamic signal not captured by the initial value alone; however, this association was not clearly reproduced in eICU and was attenuated in the endpoint-aligned MIMIC-IV analysis. Fourth, trajectory classification provided only modest incremental discrimination over the clinical model. Taken together, these findings support a cautious interpretation of Hb trajectories as data-driven, treatment-embedded longitudinal patterns rather than fixed biological phenotypes or stand-alone prognostic tools.

Previous TBI studies have commonly described Hb exposure using a single value, mean Hb, minimum Hb, or a threshold for severe anemia. Severe anemia has been associated with worse clinical outcomes (16), and improvement in cerebral tissue oxygenation after PRBC transfusion in severe TBI has not been uniform across patients (17). Peak-to-trough Hb variation has also been associated with hospital mortality (18). Longitudinal approaches are increasingly used to characterize evolving neurocritical illness: serum sodium trajectories have been studied in TBI (19), as have trajectories of the systemic immune-inflammation index (20). Related work has modeled serial GCS changes in sepsis-associated encephalopathy (21) and multivariate cerebral hemodynamic trajectories in acute brain injury (22). Our direct comparison with simpler Hb summaries provides a more qualified interpretation of the value of trajectory analysis. The overall gain in C-index was small, and the persistently low association weakened after adjustment for first-day Hb. By contrast, the rapidly declining group had a first-day Hb close to that of the high-stable group and remained associated with mortality after first-day Hb adjustment in MIMIC-IV. Thus, serial Hb data may be most informative when they reveal a change in course rather than simply restating an already low initial value.

The three trajectories occurred in different clinical contexts, but these contexts were not identical across databases. Rapidly declining Hb was associated with greater use of mechanical ventilation, vasopressors, neurosurgical or neurocritical procedures, and PRBC support, while persistently low Hb tended to occur in older patients with lower early mean arterial pressure and platelet counts. Other severity measures, including GCS, showed cohort-specific patterns. These observations reinforce that Hb trajectories are unlikely to represent isolated biological states; instead, they integrate baseline vulnerability, injury burden, measurement intensity, resuscitation, procedures, and treatment. Hb remains physiologically relevant to cerebral oxygen delivery; brain-oxygen–guided management has been tested in severe TBI (23). Mechanical ventilation can modify cerebral oxygenation and gas exchange (24), while intracranial pressure and cerebral perfusion pressure remain central targets in severe TBI care (25). Fluid therapy adds another layer because resuscitation can influence both cerebral physiology and measured Hb through hemodilution (26).

Persistently low Hb showed the most consistent transported association with mortality in the primary cross-database analyses. This group began with a substantially lower first-day Hb and remained low thereafter. The CENTER-TBI study linked lower first-week Hb with unfavorable 6-month neurological outcome and mortality (5), and a recent cohort of moderate-to-severe TBI reported an association between severe anemia and adverse outcome (16). Earlier observational work also associated anemia with worse TBI outcomes (27), reinforcing that sustained low Hb may identify a vulnerable clinical state. However, in the present study the persistently low association was attenuated after first-day Hb was entered in the same MIMIC-IV model. This finding suggests that a substantial component of the observed risk signal reflects the low starting Hb itself rather than an incremental effect attributable to class membership. Persistently low Hb therefore remains clinically important as a marker of sustained low Hb burden, but the present data do not show that this trajectory adds substantial prognostic information beyond early Hb.

The rapidly declining trajectory represented a different dynamic process. Its first-day Hb was almost identical to that of the high-stable group, followed by a marked fall during the next 24–48 h. Ongoing blood loss, extracranial injury, surgery, and repeated phlebotomy are recognized contributors to anemia after TBI (4); fluid resuscitation may further lower measured Hb through hemodilution (26). In MIMIC-IV, the rapidly declining trajectory remained associated with mortality after first-day Hb was added to the clinical model, which supports the possibility that the direction of change carries information not captured by the initial Hb value alone. This result should nevertheless be interpreted cautiously: the association was imprecise in eICU and became null in the endpoint-aligned MIMIC-IV analysis. The rapid-decline signal is therefore hypothesis-generating and requires further external evaluation rather than being treated as a reproducible prognostic phenotype.

The external assessment also illustrates the distinction between transporting a prespecified model and recovering the same latent structure *de novo*. Applying the frozen MIMIC-IV model to eICU yielded three interpretable transported patterns with broadly similar ordering and proportions. In contrast, independent three-class refitting in eICU produced one trajectory close to the MIMIC-IV rapid-decline pattern and two trajectories closer to the high-stable pattern, with no clear local analog of the persistently low class. The low label-invariant ARI (0.169) further indicated limited patient-level partition similarity. These results argue against interpreting the eICU analysis as complete replication of fixed classes. The latent classes are better viewed as model-based summaries of continuous longitudinal heterogeneity. Mixture-model methodology emphasizes that such classes are statistical summaries rather than directly observed biological entities (13), and GRoLTS reporting guidance recommends explicit attention to classification certainty and stability (14). More recent GBTM work highlights sensitivity to model specification and case mix (28), while broader trajectory-method literature similarly cautions that class boundaries may shift across samples and analytic choices (29).

From a clinical perspective, the findings favor attention to the course of Hb rather than reliance on trajectory labels themselves. Persistently low Hb should prompt consideration of pre-existing anemia, ongoing blood loss, fluid balance, renal function, nutrition, and other contributors to sustained anemia. A rapid fall from an initially preserved Hb warrants evaluation for ongoing bleeding, perioperative change, hemodilution, or other acute causes. Because the incremental discrimination of trajectory classification was modest and class boundaries were uncertain, these patterns should complement, not replace, conventional clinical assessment and established severity measures.

Clinical interpretation of Hb trajectories must also account for treatment during the same 0–120-h period. PRBC exposure differed substantially across the trajectory groups and may both respond to earlier Hb decline and modify later Hb measurements. The classes therefore represent treatment-embedded clinical courses rather than untreated natural histories. In the sensitivity analysis excluding PRBC-exposed patients, the direction of the trajectory-mortality associations was retained, indicating that the main pattern was not confined to transfused patients; this analysis does not estimate the effect of transfusion. Practice patterns for Hb targets and transfusion thresholds in acute brain injury vary across regions and specialties (30). In a randomized TBI trial, Robertson et al. (31) found no improvement in neurological recovery with a higher transfusion threshold, while TRAHT established the feasibility of comparing transfusion strategies after head trauma (32). More recent trials—HEMOTION in TBI (33) and TRAIN in acute brain injury (34)—have not yielded a single uniform message across mortality and neurological outcomes. A TBI-specific meta-analysis (35) and a broader acute-brain-injury synthesis (36) likewise suggest that treatment effects depend on the population and outcome examined. A TBI-focused review of transfusion guidance (37), together with the 2023 AABB international guideline (38), supports individualized interpretation rather than a trajectory-specific transfusion threshold. Accordingly, neither the primary analysis nor the no-PRBC sensitivity analysis should be used to infer an optimal transfusion threshold or identify a trajectory-specific treatment benefit.

Strengths of this study include the use of five consecutive 24-h windows, separation of trajectory formation from outcome follow-up by a 120-h landmark, and transport of the frozen MIMIC-IV model to a multicenter eICU cohort. We also examined several sources of uncertainty raised by longitudinal EHR data, including informative measurement and cohort selection, simpler Hb representations, posterior classification uncertainty, treatment during trajectory formation, missing covariates, and PH violations. Importantly, these analyses were used to test robustness and refine interpretation rather than to redefine the original trajectory solution after observing eICU outcomes.

This study has several limitations. First, Hb testing in routine care is informative rather than scheduled independently of clinical condition. Patients entering trajectory analysis had longer ICU stays and more frequent intensive support than those with insufficient Hb windows. Selection weighting produced similar estimates, but it can address only selection explained by measured covariates and cannot remove residual informative-measurement bias. Second, the 120-h landmark conditions the mortality analysis on survival and continued observability; in eICU, 22.4% of trajectory patients were discharged alive before the landmark. The hazard ratios therefore apply to patients who survived and remained observable at 120 h. Third, mortality endpoints differed between databases, and the endpoint-aligned analysis showed attenuation of the rapid-decline association. Fourth, independent eICU refitting did not recover the same three-class latent structure, and posterior probabilities indicated meaningful classification uncertainty. Fifth, retrospective databases lack uniform information on imaging severity, extracranial injury, bleeding volume, fluid balance, operative details, phlebotomy, and cerebral tissue oxygenation, leaving residual and time-varying confounding possible. Retrospective diagnosis and procedure coding, as well as routine clinical data capture, may also contain misclassification or measurement error. Some descriptive variables also had substantial missingness, although missingness in primary adjustment variables was limited and eICU multiple-imputation results were consistent with complete-case analyses. Finally, PRBC transfusion and other treatments occurred during trajectory formation, so the identified classes cannot be interpreted as untreated biological phenotypes, and the study cannot establish that modifying an Hb trajectory or using a particular transfusion strategy would improve outcomes.

Future studies should evaluate Hb dynamics prospectively with standardized measurement schedules and concurrent recording of bleeding, fluid administration, transfusion, surgery, cerebral perfusion, intracranial pressure, and cerebral tissue oxygenation. Repeated external assessment should determine whether rapid Hb decline or other dynamic features remain informative across different case mixes and care settings. The incremental value of dynamic Hb measures should also be tested against established TBI prognostic variables and patient-centered outcomes such as 6-month Glasgow Outcome Scale–Extended (GOSE), rather than assuming that latent-class membership itself has clinical utility.

## Conclusion

During the first 120 h after ICU admission, three data-driven Hb trajectories were identified in MIMIC-IV and transported to eICU for external assessment. Persistently low Hb showed the most consistent transported association with short-term mortality, but this association was attenuated after adjustment for first-day Hb. Rapid Hb decline retained an association with mortality after first-day Hb adjustment in MIMIC-IV, although external support was limited and the association was sensitive to endpoint definition. Trajectory classification added only modest discrimination beyond clinical variables, and independent eICU refitting did not reproduce the same three-class structure. These findings support cautious use of serial Hb patterns for describing clinical course and generating prognostic hypotheses, but they do not establish fixed biological phenotypes, causal effects, or trajectory-specific transfusion thresholds.

## Data Availability

Publicly available datasets were analyzed in this study. This data can be found at: The de-identified datasets analyzed in this study are available through PhysioNet: MIMIC-IV v3.1 (https://physionet.org/content/mimiciv/3.1/) and eICU-CRD v2.0 (https://physionet.org/content/eicu-crd/2.0/). Access is available to credentialed users subject to completion of the required training and data-use agreements. No patient-level data are redistributed with this article.
