# Supplementary material for: Early hemoglobin trajectories and short-term mortality in ICU patients with traumatic brain injury: development in MIMIC-IV and external assessment in eICU-CRD
Source: Front Neurol. 2026 Oct 6;17:1967355. doi: 10.3389/fneur.2026.1967355 (PMC13680147; doi:10.3389/fneur.2026.1967355)
Supplement: Supplementary file 1 [file Supplementary_file_1.DOCX]

Supplementary Material

Supplementary Tables S1–S16 and Supplementary Figures S1–S7.

# Supplementary Tables

**Supplementary Table S1.** Candidate latent class linear mixed models in MIMIC-IV.

| **Model** | **Classes** | **Time function** | **logLik** | **AIC** | **BIC** | **Entropy** | **Smallest class, %** | **Converged** | **Selected** |
| --- | --- | --- | --- | --- | --- | --- | --- | --- | --- |
| ng1_linear | 1 | Linear | -40760.1 | 81532.1 | 81567.0 | — | 100.0 | Yes | No |
| ng2_linear | 2 | Linear | -40654.4 | 81328.9 | 81387.0 | 0.700 | 21.9 | Yes | No |
| ng3_linear | 3 | Linear | -40610.6 | 81249.2 | 81330.5 | 0.753 | 3.7 | Yes | No |
| ng4_linear | 4 | Linear | -40589.7 | 81215.4 | 81320.0 | 0.654 | 5.8 | Yes | No |
| ng5_linear | 5 | Linear | -40563.8 | 81171.7 | 81299.5 | 0.714 | 4.0 | No | No |
| ng1_quadratic | 1 | Quadratic | -40312.9 | 80639.8 | 80680.5 | — | 100.0 | Yes | No |
| ng2_quadratic | 2 | Quadratic | -39965.3 | 79954.7 | 80024.4 | 0.769 | 13.7 | Yes | No |
| ng3_quadratic | 3 | Quadratic | -39871.4 | 79776.8 | 79875.6 | 0.652 | 16.5 | Yes | Yes |
| ng4_quadratic | 4 | Quadratic | -39833.7 | 79711.5 | 79839.3 | 0.705 | 1.6 | Yes | No |
| ng5_quadratic | 5 | Quadratic | -39803.8 | 79661.6 | 79818.5 | 0.616 | 3.1 | Yes | No |

**Note:** The selected three-class quadratic solution was retained as a balance of statistical fit, classification adequacy, and class size. Although four- and five-class quadratic models had lower BIC values, their smallest classes represented 1.6% and 3.1% of the cohort, respectively.

**Supplementary Table S2.** Posterior classification quality in MIMIC-IV and transported eICU classification.

| **Cohort** | **Trajectory** | **N** | **%** | **APPA** | **OCC** | **Median max posterior** | **Max posterior <0.70, %** | **Max posterior <0.80, %** | **Normalized entropy** |
| --- | --- | --- | --- | --- | --- | --- | --- | --- | --- |
| MIMIC-IV | Persistently low Hb | 735 | 29.8 | 0.785 | 8.59 | 0.822 | 27.5 | 45.6 | 0.6517 |
| MIMIC-IV | Relatively high and stable Hb | 1325 | 53.7 | 0.887 | 6.76 | 0.960 | 14.1 | 22.6 | 0.6517 |
| MIMIC-IV | Rapidly declining Hb | 406 | 16.5 | 0.812 | 21.89 | 0.861 | 28.6 | 42.9 | 0.6517 |
| eICU-CRD | Rapidly declining Hb | 377 | 18.0 | 0.830 | 22.29 | 0.906 | 27.6 | 36.3 | 0.6521 |
| eICU-CRD | Persistently low Hb | 537 | 25.6 | 0.773 | 9.88 | 0.805 | 32.0 | 49.2 | 0.6521 |
| eICU-CRD | Relatively high and stable Hb | 1183 | 56.4 | 0.880 | 5.69 | 0.955 | 15.5 | 23.9 | 0.6521 |

**Note:** APPA, average posterior probability of assignment; OCC, odds of correct classification. Normalized entropy was 0.6517 in MIMIC-IV and 0.6521 in eICU; both round to 0.652 at three decimals.

**Supplementary Table S3.** Distribution of Hb measurement availability in source-eligible cohorts.

| **Cohort** | **Metric** | **Category/statistic** | **N/value** |
| --- | --- | --- | --- |
| MIMIC-IV | Observed Hb windows | 0 | 141 |
| MIMIC-IV | Observed Hb windows | 1 | 446 |
| MIMIC-IV | Observed Hb windows | 2 | 491 |
| MIMIC-IV | Observed Hb windows | 3 | 518 |
| MIMIC-IV | Observed Hb windows | 4 | 649 |
| MIMIC-IV | Observed Hb windows | 5 | 1299 |
| MIMIC-IV | Total Hb measurements | median | 5 |
| MIMIC-IV | Total Hb measurements | Q1 | 3 |
| MIMIC-IV | Total Hb measurements | Q3 | 6 |
| eICU-CRD | Observed Hb windows | 0 | 437 |
| eICU-CRD | Observed Hb windows | 1 | 1079 |
| eICU-CRD | Observed Hb windows | 2 | 767 |
| eICU-CRD | Observed Hb windows | 3 | 633 |
| eICU-CRD | Observed Hb windows | 4 | 489 |
| eICU-CRD | Observed Hb windows | 5 | 975 |
| eICU-CRD | Total Hb measurements | median | 3 |
| eICU-CRD | Total Hb measurements | Q1 | 1 |
| eICU-CRD | Total Hb measurements | Q3 | 5 |

**Note:** Observed windows refer to the five prespecified 24-h windows from 0 to 120 h. For total Hb measurements, median, Q1, and Q3 are shown.

**Supplementary Table S4.** MIMIC-IV source-eligible, trajectory-included, and trajectory-excluded patients.

| **Variable** | **Source eligible** | **Trajectory excluded** | **Trajectory included** |
| --- | --- | --- | --- |
| N | 3544 | 1078 | 2466 |
| Age, years | 67.9 [IQR 49.6-82.3] | 63.6 [IQR 41.1-82.1] | 69.3 [IQR 52.7-82.4] |
| Male sex | 2207 (62.3%) | 661 (61.3%) | 1546 (62.7%) |
| GCS | 14.0 [IQR 13.0-15.0] | 15.0 [IQR 14.0-15.0] | 14.0 [IQR 13.0-15.0] |
| Mechanical ventilation within 24 h | 1326 (37.4%) | 316 (29.3%) | 1010 (41.0%) |
| Vasopressor within 24 h | 414 (11.7%) | 125 (11.6%) | 289 (11.7%) |
| Non-CNS SOFA | 2.0 [IQR 1.0-4.0] | 1.0 [IQR 1.0-3.0] | 2.0 [IQR 1.0-4.0] |
| ICU length of stay, days | 2.0 [IQR 1.0-4.4] | 1.0 [IQR 0.7-1.6] | 2.9 [IQR 1.7-6.3] |
| Hospital mortality | 475 (13.4%) | 253 (23.5%) | 222 (9.0%) |
| Documented PRBC within 120 h | 450 (12.7%) | 62 (5.8%) | 388 (15.7%) |
| Total Hb measurements | 5.0 [IQR 3.0-6.0] | 2.0 [IQR 1.0-2.0] | 5.0 [IQR 4.0-7.0] |
| Observed Hb windows | 4.0 [IQR 2.0-5.0] | 1.0 [IQR 1.0-2.0] | 5.0 [IQR 4.0-5.0] |

**Note:** Trajectory inclusion required Hb in at least three of five 24-h windows. Values are median [IQR] or n (%).

**Supplementary Table S5.** eICU-CRD source-eligible, trajectory-included, and trajectory-excluded patients.

| **Variable** | **Source eligible** | **Trajectory excluded** | **Trajectory included** |
| --- | --- | --- | --- |
| N | 4380 | 2283 | 2097 |
| Age, years | 66.0 [IQR 47.0-80.0] | 68.0 [IQR 48.0-81.0] | 64.0 [IQR 46.0-79.0] |
| Male sex | 2615 (59.7%) | 1321 (57.9%) | 1294 (61.7%) |
| GCS | 14.0 [IQR 9.0-15.0] | 14.0 [IQR 12.0-15.0] | 13.0 [IQR 7.0-15.0] |
| Mechanical ventilation within 24 h | 1478 (33.7%) | 519 (22.7%) | 959 (45.7%) |
| Vasopressor within 24 h | 259 (5.9%) | 84 (3.7%) | 175 (8.3%) |
| APACHE score | 48.0 [IQR 35.0-66.0] | 43.0 [IQR 31.0-59.0] | 53.0 [IQR 39.8-71.0] |
| ICU length of stay, days | 1.8 [IQR 0.9-3.9] | 1.0 [IQR 0.7-1.7] | 3.8 [IQR 2.1-7.6] |
| Hospital mortality | 519 (11.8%) | 307 (13.4%) | 212 (10.1%) |
| Documented PRBC within 120 h | 152 (3.5%) | 40 (1.8%) | 112 (5.3%) |
| Total Hb measurements | 3.0 [IQR 1.0-5.0] | 1.0 [IQR 1.0-2.0] | 5.0 [IQR 4.0-6.0] |
| Observed Hb windows | 2.0 [IQR 1.0-4.0] | 1.0 [IQR 1.0-2.0] | 4.0 [IQR 3.0-5.0] |

**Note:** Trajectory inclusion required Hb in at least three of five 24-h windows. Values are median [IQR] or n (%).

**Supplementary Table S6.** Bootstrap stability of the selected MIMIC-IV three-class solution.

| **Metric** | **Median / count** | **IQR** | **Range** |
| --- | --- | --- | --- |
| Successful refits | 100/100 |  |  |
| Adjusted Rand index | 0.86 | 0.79–0.90 | 0.34–0.96 |
| Patient-level class concordance | 0.95 | Not prespecified for reporting | 0.74–0.99 |

**Note:** Bootstrap refitting was performed at the patient level. The observed range indicates that classification was generally stable but not invariant across resamples.

**Supplementary Table S7A.** Proportional-hazards diagnostics for MIMIC-IV Model 3.

| **Term** | **Chi-square** | **df** | **P value** |
| --- | --- | --- | --- |
| Trajectory class | 0.328 | 2 | 0.849 |
| Age | 3.992 | 1 | 0.046 |
| Sex | 0.978 | 1 | 0.323 |
| GCS | 0.072 | 1 | 0.788 |
| Non-CNS SOFA | 0.019 | 1 | 0.890 |
| Mechanical ventilation within 24 h | 6.176 | 1 | 0.013 |
| Vasopressor within 24 h | 0.343 | 1 | 0.558 |
| Neurosurgery/neurocritical procedure | 5.087 | 1 | 0.024 |
| Charlson Comorbidity Index | 14.817 | 1 | <0.001 |
| Global | 26.686 | 10 | 0.003 |

**Note:** Schoenfeld-residual diagnostics. The trajectory term did not violate the PH assumption; the global signal was driven by selected adjustment covariates.

**Supplementary Table S7B.** PH-corrected sensitivity models.

| **Analysis** | **Term** | **HR (95% CI)** | **P value** | **N** | **Events** |
| --- | --- | --- | --- | --- | --- |
| Original Model 3 diagnostic refit | Rapidly declining Hb | 1.74 (1.18–2.57) | 0.005 | 2404 | 260 |
| Original Model 3 diagnostic refit | Persistently low Hb | 1.57 (1.19–2.08) | 0.001 | 2404 | 260 |
| PH-corrected Model 3 | Rapidly declining Hb | 1.74 (1.18–2.56) | 0.005 | 2404 | 260 |
| PH-corrected Model 3 | Persistently low Hb | 1.57 (1.19–2.08) | 0.002 | 2404 | 260 |
| eICU stratified PH-corrected Model 3 | Rapidly declining Hb | 1.32 (0.85–2.04) | 0.218 | 1503 | 131 |
| eICU stratified PH-corrected Model 3 | Persistently low Hb | 1.67 (1.09–2.57) | 0.018 | 1503 | 131 |

**Note:** For MIMIC-IV, binary covariates with nonproportionality were stratified and continuous covariates were allowed time-varying effects when indicated. The eICU-CRD estimates were obtained after stratifying the Cox model for covariates that showed evidence of nonproportional hazards. The directions of the trajectory associations were unchanged, and the global proportional-hazards test no longer suggested meaningful violation (P = 0.784).

**Supplementary Table S8.** MIMIC-IV analysis aligned to the eICU in-hospital mortality endpoint.

| **Trajectory** | **HR (95% CI)** | **Interpretation** |
| --- | --- | --- |
| Relatively high and stable Hb | Reference |  |
| Rapidly declining Hb | 1.00 (0.61–1.64) | Direction attenuated to null |
| Persistently low Hb | 1.57 (1.10–2.24) | Association retained |

**Note:** The risk set began at 120 h. Hospital death was the event; live discharge was treated as censoring, matching the eICU endpoint framework.

**Supplementary Table S9A.** Predicted Hb profiles from the independent eICU three-class refit.

| **Local class** | **0–24 h** | **24–48 h** | **48–72 h** | **72–96 h** | **96–120 h** |
| --- | --- | --- | --- | --- | --- |
| Local class 1 | 119.6 | 99.7 | 88.9 | 87.3 | 94.7 |
| Local class 2 | 115.2 | 120.6 | 122.6 | 121.3 | 116.6 |
| Local class 3 | 113.0 | 107.9 | 105.4 | 105.3 | 107.8 |

**Note:** No distinct local class closely resembled the persistently low MIMIC-IV trajectory. Local class 1 most closely resembled rapid decline, whereas local classes 2 and 3 were closer to high/stable patterns by curve shape.

**Supplementary Table S9B.** Partition similarity between transported and independently refitted eICU classifications.

| **Metric** | **Class** | **Denominator N** | **Concordant N** | **Value** |
| --- | --- | --- | --- | --- |
| Best-match patient agreement | Overall | 2097 | 1264 | 0.603 |
| Adjusted Rand index | Overall | 2097 |  | 0.169 |
| Descriptive class-specific curve-matched agreement | Rapidly declining Hb | 377 | 141.0 | 0.374 |
| Descriptive class-specific curve-matched agreement | Relatively high and stable Hb | 1183 | 179.0 | 0.151 |
| Descriptive class-specific curve-matched agreement | Persistently low Hb | 537 | 0.0 | 0.000 |

**Note:** Labels from the eICU de novo refit were not matched mechanically by numeric class identifiers. Descriptive curve matching was based on the five 24-h Hb profiles: local class 1 showed a rapidly declining pattern, whereas local classes 2 and 3 were closer to the relatively high and stable pattern; no stable local class fully corresponded to the MIMIC persistently low pattern. Best-match patient agreement was 60.3%, and the adjusted Rand index was 0.169. Because the adjusted Rand index is invariant to label permutation, it is the preferred metric for patient-level partition similarity.

**Supplementary Table S10.** Timing of first documented PRBC exposure during trajectory formation.

| **Cohort** | **Trajectory** | **First-PRBC window** | **N** | **First PRBC time from ICU admission, h, median (IQR)** |
| --- | --- | --- | --- | --- |
| MIMIC-IV | Persistently low Hb | 0–24 h | 169 | 5.4 (2.6–11.1) |
| MIMIC-IV | Rapidly declining Hb | 0–24 h | 65 | 2.9 (1.4–5.9) |
| MIMIC-IV | Persistently low Hb | 24–48 h | 45 | 31.6 (28.0–39.4) |
| MIMIC-IV | Rapidly declining Hb | 72–96 h | 7 | 86.1 (79.3–89.4) |
| MIMIC-IV | Persistently low Hb | 48–72 h | 17 | 59.2 (51.9–61.1) |
| MIMIC-IV | Rapidly declining Hb | 48–72 h | 17 | 57.2 (53.0–62.5) |
| MIMIC-IV | Relatively high and stable Hb | 0–24 h | 28 | 3.3 (1.5–6.0) |
| MIMIC-IV | Relatively high and stable Hb | 48–72 h | 1 | 52.9 (52.9–52.9) |
| MIMIC-IV | Persistently low Hb | 72–96 h | 6 | 83.2 (76.0–91.2) |
| MIMIC-IV | Rapidly declining Hb | 24–48 h | 18 | 36.6 (29.6–41.4) |
| MIMIC-IV | Relatively high and stable Hb | 24–48 h | 5 | 41.7 (29.1–45.1) |
| MIMIC-IV | Persistently low Hb | 96–120 h | 5 | 115.4 (104.2–118.9) |
| MIMIC-IV | Rapidly declining Hb | 96–120 h | 5 | 113.0 (111.7–118.4) |
| eICU-CRD | Rapidly declining Hb | 0–24 h | 21 | 1.5 (0.7–4.0) |
| eICU-CRD | Rapidly declining Hb | 72–96 h | 5 | 89.6 (85.4–92.3) |
| eICU-CRD | Persistently low Hb | 0–24 h | 49 | 1.8 (0.6–5.7) |
| eICU-CRD | Rapidly declining Hb | 24–48 h | 1 | 46.8 (46.8–46.8) |
| eICU-CRD | Persistently low Hb | 48–72 h | 6 | 55.1 (54.9–55.5) |
| eICU-CRD | Persistently low Hb | 24–48 h | 9 | 33.5 (30.9–37.5) |
| eICU-CRD | Relatively high and stable Hb | 0–24 h | 11 | 2.3 (0.7–4.5) |
| eICU-CRD | Relatively high and stable Hb | 24–48 h | 2 | 35.3 (32.0–38.6) |
| eICU-CRD | Persistently low Hb | 96–120 h | 2 | 98.0 (97.2–98.8) |
| eICU-CRD | Persistently low Hb | 72–96 h | 2 | 85.2 (82.5–87.9) |
| eICU-CRD | Rapidly declining Hb | 48–72 h | 2 | 58.2 (53.3–63.2) |
| eICU-CRD | Relatively high and stable Hb | 48–72 h | 1 | 52.8 (52.8–52.8) |
| eICU-CRD | Rapidly declining Hb | 96–120 h | 1 | 97.8 (97.8–97.8) |

**Note:** This table is descriptive and does not estimate the causal effect of transfusion.

**Supplementary Table S11.** Inverse-probability-of-selection weighted sensitivity analyses.

| **Cohort** | **Analysis** | **Trajectory term** | **HR (95% CI)** | **P value** | **N** | **Events** | **Weight median (Q1–Q3)** | **Weight P1–P99** | **Effective sample size** |
| --- | --- | --- | --- | --- | --- | --- | --- | --- | --- |
| MIMIC-IV | Stabilized selection IPW | Rapidly declining Hb | 1.75 (1.20–2.56) | 0.004 | 2404 | 260 | 0.970 (0.869–1.097) | 0.738–1.452 | 2338.4 |
| MIMIC-IV | Stabilized selection IPW | Persistently low Hb | 1.57 (1.18–2.09) | 0.002 | 2404 | 260 | 0.970 (0.869–1.097) | 0.738–1.452 | 2338.4 |
| MIMIC-IV | Winsorized selection IPW (P1/P99) | Rapidly declining Hb | 1.75 (1.20–2.56) | 0.004 | 2404 | 260 | 0.970 (0.869–1.097) | 0.738–1.452 | 2339.6 |
| MIMIC-IV | Winsorized selection IPW (P1/P99) | Persistently low Hb | 1.57 (1.18–2.09) | 0.002 | 2404 | 260 | 0.970 (0.869–1.097) | 0.738–1.452 | 2339.6 |
| eICU-CRD | Stabilized selection IPW | Rapidly declining Hb | 1.26 (0.81–1.96) | 0.297 | 1503 | 131 | 0.955 (0.722–1.159) | 0.634–1.793 | 1390.5 |
| eICU-CRD | Stabilized selection IPW | Persistently low Hb | 1.59 (1.04–2.44) | 0.033 | 1503 | 131 | 0.955 (0.722–1.159) | 0.634–1.793 | 1390.5 |
| eICU-CRD | Winsorized selection IPW (P1/P99) | Rapidly declining Hb | 1.27 (0.82–1.96) | 0.293 | 1503 | 131 | 0.955 (0.722–1.159) | 0.635–1.792 | 1394.9 |
| eICU-CRD | Winsorized selection IPW (P1/P99) | Persistently low Hb | 1.59 (1.04–2.44) | 0.032 | 1503 | 131 | 0.955 (0.722–1.159) | 0.635–1.792 | 1394.9 |

**Note:** The selection model estimated inclusion in the trajectory cohort. MIMIC-IV covariates were age, sex, GCS, non-CNS SOFA, Charlson Comorbidity Index, mechanical ventilation within 24 h, and vasopressor use within 24 h; eICU-CRD covariates were age, sex, GCS, APACHE score, past-history item count, mechanical ventilation within 24 h, and vasopressor use within 24 h. Missing continuous covariates were median-filled with corresponding missingness indicators solely for the selection model, and missing categorical values were retained as a separate level. Stabilized weights used the marginal inclusion probability as the numerator and the individual predicted inclusion probability as the denominator; predicted probabilities were clipped to 0.01–0.99. Extreme weights were winsorized at P1/P99 in the designated sensitivity analysis. This analysis assessed selection related to Hb measurement availability and the subsequent 120-h landmark analytic pathway.

**Supplementary Table S12A.** Attrition before the 120-h landmark.

| **Cohort** | **Landmark status/reason** | **N** | **%** | **Trajectory cohort denominator** |
| --- | --- | --- | --- | --- |
| MIMIC-IV | included | 2409 | 97.7 | 2466 |
| MIMIC-IV | died before 120 h | 57 | 2.3 | 2466 |
| eICU-CRD | included | 1557 | 74.2 | 2097 |
| eICU-CRD | live discharge before 120 h | 469 | 22.4 | 2097 |
| eICU-CRD | died before 120 h | 71 | 3.4 | 2097 |

**Note:** The larger eICU attrition was driven predominantly by live discharge before 120 h.

**Supplementary Table S12B.** MIMIC-IV landmark-included versus landmark-excluded patients.

| **Variable** | **Landmark excluded** | **Landmark included** |
| --- | --- | --- |
| Age, years | 71.9 [IQR 54.8-87.7] | 69.2 [IQR 52.7-82.3] |
| Documented PRBC within 120 h | 15 (26.3%) | 373 (15.5%) |
| GCS | 15.0 [IQR 10.0-15.0] | 14.0 [IQR 13.0-15.0] |
| ICU length of stay, days | 3.7 [IQR 3.0-4.2] | 2.9 [IQR 1.6-6.5] |
| Male sex | 36 (63.2%) | 1510 (62.7%) |
| Mechanical ventilation within 24 h | 41 (71.9%) | 969 (40.2%) |
| Non-CNS SOFA | 3.0 [IQR 2.0-6.0] | 2.0 [IQR 1.0-4.0] |
| Trajectory: High/stable | 11 (19.3%) | 1314 (54.5%) |
| Trajectory: Persistently low | 27 (47.4%) | 708 (29.4%) |
| Trajectory: Rapid decline | 19 (33.3%) | 387 (16.1%) |
| Vasopressor within 24 h | 23 (40.4%) | 265 (11.0%) |

**Note:** Values are median [IQR] or n (%). The post-landmark HRs apply to patients who survived and remained observable at 120 h.

**Supplementary Table S12C.** eICU-CRD landmark-included versus landmark-excluded patients.

| **Variable** | **Landmark excluded** | **Landmark included** |
| --- | --- | --- |
| APACHE score | 48.0 [IQR 37.0-61.0] | 55.0 [IQR 40.0-73.0] |
| Age, years | 69.0 [IQR 50.0-81.0] | 63.0 [IQR 44.0-79.0] |
| Documented PRBC within 120 h | 13 (2.4%) | 99 (6.4%) |
| GCS | 14.0 [IQR 12.0-15.0] | 12.0 [IQR 7.0-14.0] |
| ICU length of stay, days | 2.1 [IQR 1.4-3.0] | 5.2 [IQR 2.9-10.2] |
| Male sex | 299 (55.4%) | 995 (63.9%) |
| Mechanical ventilation within 24 h | 136 (25.2%) | 823 (52.9%) |
| Trajectory: High/stable | 381 (70.6%) | 802 (51.5%) |
| Trajectory: Persistently low | 105 (19.4%) | 432 (27.7%) |
| Trajectory: Rapid decline | 54 (10.0%) | 323 (20.7%) |
| Vasopressor within 24 h | 37 (6.9%) | 138 (8.9%) |

**Note:** Values are median [IQR] or n (%). The post-landmark HRs apply to patients who survived and remained observable at 120 h.

**Supplementary Table S13A.** Incremental prognostic information in MIMIC-IV on a common analysis sample.

| **Model** | **N** | **Events** | **AIC** | **C-index** | **ΔC-index, bootstrap median (95% interval)** |
| --- | --- | --- | --- | --- | --- |
| Clinical model | 2378 | 255 | 3681.8 | 0.786 | 0.0000 (0.0000 to 0.0000) |
| Clinical + first-day Hb | 2378 | 255 | 3676.0 | 0.789 | 0.0018 (-0.0007 to 0.0082) |
| Clinical + 0–120 h mean Hb | 2378 | 255 | 3672.2 | 0.790 | 0.0033 (-0.0004 to 0.0096) |
| Clinical + 0–120 h minimum Hb | 2378 | 255 | 3670.2 | 0.791 | 0.0043 (-0.0001 to 0.0117) |
| Clinical + linear Hb slope | 2378 | 255 | 3683.8 | 0.786 | 0.0001 (-0.0012 to 0.0027) |
| Clinical + trajectory class | 2378 | 255 | 3672.1 | 0.792 | 0.0054 (0.0005 to 0.0123) |
| Clinical + first-day Hb + trajectory class | 2378 | 255 | 3672.1 | 0.792 | 0.0062 (0.0006 to 0.0124) |

**Note:** All models used the same clinical covariate set and common sample. ΔC-index is relative to Model A and is based on 200 patient-level bootstrap resamples.

**Supplementary Table S13B.** Incremental prognostic information in eICU-CRD on a common analysis sample.

| **Model** | **N** | **Events** | **AIC** | **C-index** | **ΔC-index, bootstrap median (95% interval)** |
| --- | --- | --- | --- | --- | --- |
| Clinical model | 1429 | 128 | 1627.8 | 0.708 | 0.0000 (0.0000 to 0.0000) |
| Clinical + first-day Hb | 1429 | 128 | 1629.0 | 0.707 | -0.0002 (-0.0072 to 0.0092) |
| Clinical + 0–120 h mean Hb | 1429 | 128 | 1628.2 | 0.708 | 0.0003 (-0.0042 to 0.0075) |
| Clinical + 0–120 h minimum Hb | 1429 | 128 | 1627.7 | 0.710 | 0.0011 (-0.0023 to 0.0108) |
| Clinical + linear Hb slope | 1429 | 128 | 1629.2 | 0.711 | 0.0012 (-0.0026 to 0.0166) |
| Clinical + trajectory class | 1429 | 128 | 1628.0 | 0.714 | 0.0052 (-0.0021 to 0.0206) |
| Clinical + first-day Hb + trajectory class | 1429 | 128 | 1629.7 | 0.717 | 0.0099 (-0.0020 to 0.0296) |

**Note:** All models used the same external clinical covariate set and common sample. Bootstrap intervals for ΔC-index crossed zero.

**Supplementary Table S13C.** First-day Hb and trajectory class entered jointly.

| **Cohort** | **Term** | **HR (95% CI)** | **P value** | **N** | **Events** |
| --- | --- | --- | --- | --- | --- |
| MIMIC-IV | First-day Hb (per 1 g/L) | 0.99 (0.98–1.00) | 0.158 | 2378 | 255 |
| MIMIC-IV | Rapidly declining Hb | 1.77 (1.20–2.62) | 0.004 | 2378 | 255 |
| MIMIC-IV | Persistently low Hb | 1.27 (0.83–1.93) | 0.268 | 2378 | 255 |
| eICU-CRD | First-day Hb (per 1 g/L) | 1.00 (0.99–1.02) | 0.571 | 1429 | 128 |
| eICU-CRD | Rapidly declining Hb | 1.21 (0.77–1.90) | 0.404 | 1429 | 128 |
| eICU-CRD | Persistently low Hb | 1.69 (0.94–3.03) | 0.081 | 1429 | 128 |

**Note:** In MIMIC-IV, the persistently low trajectory association attenuated after adjustment for first-day Hb, whereas rapid decline retained an association with mortality.

**Supplementary Table S14A.** High-certainty class-assignment sensitivity analyses.

| **Cohort** | **Posterior threshold** | **Trajectory term** | **HR (95% CI)** | **P value** | **N** | **Events** | **Class counts** |
| --- | --- | --- | --- | --- | --- | --- | --- |
| MIMIC-IV | 0.7 | Rapidly declining Hb | 1.95 (1.20–3.17) | 0.007 | 1910 | 198 | Persistently low=510; High/stable=1127; Rapid decline=273 |
| MIMIC-IV | 0.7 | Persistently low Hb | 1.60 (1.16–2.20) | 0.004 | 1910 | 198 | Persistently low=510; High/stable=1127; Rapid decline=273 |
| MIMIC-IV | 0.8 | Rapidly declining Hb | 1.76 (1.00–3.09) | 0.049 | 1625 | 169 | Persistently low=388; High/stable=1018; Rapid decline=219 |
| MIMIC-IV | 0.8 | Persistently low Hb | 1.56 (1.11–2.20) | 0.011 | 1625 | 169 | Persistently low=388; High/stable=1018; Rapid decline=219 |
| eICU-CRD | 0.7 | Rapidly declining Hb | 1.27 (0.71–2.28) | 0.413 | 1150 | 98 | Rapid decline=229; High/stable=646; Persistently low=275 |
| eICU-CRD | 0.7 | Persistently low Hb | 1.77 (1.14–2.73) | 0.010 | 1150 | 98 | Rapid decline=229; High/stable=646; Persistently low=275 |
| eICU-CRD | 0.8 | Rapidly declining Hb | 1.21 (0.66–2.22) | 0.538 | 971 | 89 | High/stable=572; Rapid decline=200; Persistently low=199 |
| eICU-CRD | 0.8 | Persistently low Hb | 2.22 (1.41–3.51) | <0.001 | 971 | 89 | High/stable=572; Rapid decline=200; Persistently low=199 |

**Note:** Models were repeated after restricting to patients with maximum posterior probability ≥0.70 or ≥0.80.

**Supplementary Table S14B.** Pseudo-class assignment sensitivity analysis.

| **Cohort** | **Trajectory** | **Median HR (central 95% of draws)** | **Draws with HR >1** | **Successful draws** | **N** | **Events** |
| --- | --- | --- | --- | --- | --- | --- |
| MIMIC-IV | Rapid decline | 1.39 (1.13–1.73) | 99% | 100 | 2404 | 260 |
| MIMIC-IV | Persistently low | 1.41 (1.16–1.66) | 100% | 100 | 2404 | 260 |
| eICU-CRD | Rapid decline | 1.10 (0.84–1.55) | 76% | 100 | 1503 | 131 |
| eICU-CRD | Persistently low | 1.42 (1.08–1.89) | 99% | 100 | 1503 | 131 |

**Note:** Class membership was sampled 100 times from each patient’s posterior-probability vector, and the same adjusted Cox model was refitted in each draw.

**Supplementary Table S15.** Sensitivity analysis excluding patients with documented PRBC exposure during 0–120 h.

| **Cohort** | **Trajectory term** | **HR (95% CI)** | **P value** | **N** | **Events** | **Class counts** |
| --- | --- | --- | --- | --- | --- | --- |
| MIMIC-IV | Rapidly declining Hb | 1.87 (1.22–2.88) | 0.004 | 2031 | 208 | Persistently low=475; High/stable=1279; Rapid decline=277 |
| MIMIC-IV | Persistently low Hb | 1.73 (1.27–2.35) | <0.001 | 2031 | 208 | Persistently low=475; High/stable=1279; Rapid decline=277 |
| eICU-CRD | Rapidly declining Hb | 1.39 (0.89–2.17) | 0.152 | 1408 | 112 | High/stable=765; Rapid decline=284; Persistently low=359 |
| eICU-CRD | Persistently low Hb | 1.50 (0.94–2.40) | 0.090 | 1408 | 112 | High/stable=765; Rapid decline=284; Persistently low=359 |

**Note:** This analysis assesses whether the trajectory–mortality associations were confined to transfused patients; it does not estimate a transfusion treatment effect.

**Supplementary Table S16A.** Missingness of primary eICU Model 3 covariates at the 120-h landmark.

| **Variable** | **N** | **Missing N** | **Missing, %** |
| --- | --- | --- | --- |
| Age | 1557 | 0 | 0.00 |
| Sex | 1557 | 1 | 0.06 |
| GCS | 1557 | 53 | 3.40 |
| Mechanical ventilation within 24 h | 1557 | 0 | 0.00 |
| Vasopressor within 24 h | 1557 | 0 | 0.00 |

**Note:** GCS was the main source of missingness among primary adjustment covariates.

**Supplementary Table S16B.** eICU multiple-imputation sensitivity analysis.

| **Analysis** | **Trajectory term** | **HR (95% CI)** | **P value** | **N** | **Events** |
| --- | --- | --- | --- | --- | --- |
| Complete-case Model 3 | Rapidly declining Hb | 1.24 (0.80–1.92) | 0.340 | 1503 | 131 |
| Complete-case Model 3 | Persistently low Hb | 1.58 (1.03–2.43) | 0.036 | 1503 | 131 |
| MICE m=20; Rubin pooled hospital-cluster robust Cox | Rapidly declining Hb | 1.33 (0.85–2.07) | 0.217 | 1557 | 135 |
| MICE m=20; Rubin pooled hospital-cluster robust Cox | Persistently low Hb | 1.63 (1.06–2.51) | 0.025 | 1557 | 135 |

**Note:** Multiple imputation by chained equations used m=20 datasets. Hospital-cluster robust Cox estimates were combined using Rubin’s rules.

# Supplementary Figures

**Supplementary Figure S1.** Distribution of observed 24-h Hb windows in the MIMIC-IV and eICU-CRD source-eligible cohorts. The trajectory cohort required observations in at least three of five windows.

**Supplementary Figure S2.** Distribution summary of stabilized inverse-probability-of-selection weights. Thin lines show P1–P99, thick lines show the IQR, and points show medians.

**Supplementary Figure S3.** Incremental Harrell C-index after adding simple Hb summaries or trajectory information to the clinical model. Points show bootstrap median ΔC-index and whiskers show the bootstrap 95% interval.

**Supplementary Figure S4.** Proportions of the three MIMIC-IV-derived Hb trajectory classes in the development cohort and after transport of the frozen model to eICU-CRD.

**Supplementary Figure S5.** Predicted Hb profiles from the frozen MIMIC-IV model and the independent eICU three-class refit. The local refit produced one rapid-decline-like trajectory and two higher-level trajectories; no distinct local class closely resembled the persistently low MIMIC-IV trajectory.

**Supplementary Figure S6.** Timing window of first documented PRBC exposure during the 0–120-h trajectory-construction period, by transported trajectory class and cohort.

**Supplementary Figure S7.** MIMIC-IV endpoint-aligned sensitivity analysis using in-hospital mortality with live-discharge censoring to match the eICU outcome framework. The relatively high and stable trajectory is the reference.
